# Exon Array Analysis of Head and Neck Cancers Identifies a Hypoxia Related Splice Variant of *LAMA3* Associated with a Poor Prognosis

**DOI:** 10.1371/journal.pcbi.1000571

**Published:** 2009-11-20

**Authors:** Carla S. Moller-Levet, Guy N. J. Betts, Adrian L. Harris, Jarrod J. Homer, Catharine M. L. West, Crispin J. Miller

**Affiliations:** 1 Applied Computational Biology and Bioinformatics Group, Cancer Research UK Paterson Institute for Cancer Research, The University of Manchester, Christie Hospital, Manchester, United Kingdom; 2 Translational Radiobiology Group, School of Cancer and Imaging Sciences, The University of Manchester, Christie Hospital, Manchester, United Kingdom; 3 Cancer Research UK Molecular Oncology Laboratories, Weatherall Institute of Molecular Medicine, John Radcliffe Hospital, Oxford, United Kingdom; 4 Department of Otolaryngology-Head and Neck Surgery, The University of Manchester Academic Health Science Centre, Manchester Royal Infirmary, Manchester, United Kingdom; Lilly Singapore Centre for Drug Discovery, Singapore

## Abstract

The identification of alternatively spliced transcript variants specific to particular biological processes in tumours should increase our understanding of cancer. Hypoxia is an important factor in cancer biology, and associated splice variants may present new markers to help with planning treatment. A method was developed to analyse alternative splicing in exon array data, using probeset multiplicity to identify genes with changes in expression across their loci, and a combination of the splicing index and a new metric based on the variation of reliability weighted fold changes to detect changes in the splicing patterns. The approach was validated on a cancer/normal sample dataset in which alternative splicing events had been confirmed using RT-PCR. We then analysed ten head and neck squamous cell carcinomas using exon arrays and identified differentially expressed splice variants in five samples with high versus five with low levels of hypoxia-associated genes. The analysis identified a splice variant of *LAMA3* (Laminin α 3), LAMA3-A, known to be involved in tumour cell invasion and progression. The full-length transcript of the gene (LAMA3-B) did not appear to be hypoxia-associated. The results were confirmed using qualitative RT-PCR. In a series of 59 prospectively collected head and neck tumours, expression of LAMA3-A had prognostic significance whereas LAMA3-B did not. This work illustrates the potential for alternatively spliced transcripts to act as biomarkers of disease prognosis with improved specificity for particular tissues or conditions over assays which do not discriminate between splice variants.

## Author Summary

Alternative splicing is the process by which cells express a set of different, but related, transcripts from a single gene. When translated, each transcript results in a different protein, resulting in additional cellular complexity. Affymetrix Exon microarrays, which feature multiple probesets targeting different locations throughout each gene, allow the changes in transcription that result from alternative splicing to be investigated in a single genome-wide assay. In addition, the increased number of probesets targeting each gene offers the potential to combine signals in order to increase statistical power, allowing smaller changes to be detected reliably. We developed a novel algorithm to exploit both these aspects of exon arrays and applied it to tumour hypoxia in clinical samples. Our method identified 4 potential transcript variants upregulated in hypoxic cancers, including a splice variant of the Laminin alpha 3 gene, which we were then able to validate by other methods. On further investigation, we found that expression of this particular isoform in head and neck cancers was a strong adverse prognostic factor for survival following primary surgical treatment. This shows that exon arrays can be used to identify clinically relevant splicing events with potential utility as prognostic biomarkers.

## Introduction

Alternative splicing is the process by which cells can selectively include different sections of pre-mRNA during RNA processing. If these transcripts are translated, this results in a set of closely related, but different, proteins expressed from a single locus [1],[2]. Alternative splicing is prevalent (the majority of human genes are alternatively spliced, with an average of about 5.4 transcripts per gene [3]), and tightly regulated. It is a key player in many molecular pathways, and is known to be involved in many of the ‘hallmark’ processes of cancer [4], including resistance to apoptosis, invasion, angiogenesis and differentiation [5].

Until recently, a lack of appropriate tools has made it impossible to perform routine global surveys of alternative splicing, making it relatively understudied. Recently, a set of exon microarrays has been developed by Affymetrix. These feature probesets targeting at intervals throughout each transcript, rather than simply at the 3′ end interrogated by most other arrays. This enables the assembly, *in silico*, of expression levels across genes providing a more complete representation of transcription for each gene, and allowing the identification of loci where there are changes in the splicing pattern across experimental samples. Another useful consequence of the increased resolution of the arrays is that since most transcripts are targeted by multiple probesets, their signals can be combined in order to increase statistical power [6]. This leads to the identification of differentially expressed genes with smaller effects sizes than can be found using other, less comprehensive platforms. However, this increased performance is not without additional challenges, since detailed analysis of the arrays requires annotation describing the known relationships between genes, transcripts and exons, and the ability to combine this with appropriate statistics [7]–[9].

Here we use Affymetrix Exon 1.0ST arrays to study hypoxia in human cancers. Hypoxia can lead to an altered, invasive tumour phenotype through wide ranging changes in gene transcription within a cell. Perhaps the best known mediator of this is hypoxia inducible factor 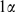 (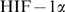), a transcription factor that regulates the expression of many tumorigenic genes involved in a wide range of cellular processes, including angiogenesis, cell proliferation, apoptosis and cell migration [10]. Several well known 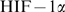 regulated (and cancer associated) genes (e.g. *VEGFA* [11], *CA9* [12]) are already known to be alternatively spliced, although the relationship between hypoxia, differential transcript expression, alternative splicing, and tumour phenotype has yet to be fully determined. Given the ubiquity of alternative splicing, it is likely that there are many more such events to be discovered.

To date, most published metrics used to analyze alternative splicing compute an overall gene level summary that is used as a baseline against which the behaviour of its constituent exons can be compared. The most popular metric is the splicing index [2],[13],[14], which aims to identify probesets that have different inclusion rates (relative to the gene level) between two sample groups. MIDAS is an extension of the splicing index that uses ANOVA instead of a t-test to evaluate significance, allowing comparisons between multiple sample groups. In FIRMA [15], the popular linear model Robust Multichip Analysis (RMA) (used for normalization and summarization of probeset intensities) is fitted to each gene in order to estimate overall expression level in each sample, while the median of the residuals in an exon is used to generate a summary statistic of each exon's alternative splicing. The method was developed to evaluate situations where there are neither replicates nor pre-defined groups. MADS [16] calculates splicing indices and p-values of individual probes separately, prior to summarization at probeset level. The Pattern-based Correlation (PAC) [17],[18] algorithm is based on the correlation across samples between exon expression levels and the overall gene expression level. PAC is limited by the number of samples, since it works best when there are enough differentially spliced samples to significantly weaken the correlation between gene and exon. Genes, exons or probesets scoring highly in an algorithm/metric (and usually accompanied by a low probability score) are identified as promising candidates of alternative splicing events and are suitable for further tests. As discussed elsewhere [15], alternative splicing is an analogue process, with no threshold above which alternative splicing can be said to occur. Results are therefore usually reported as a ranked list.

We used exon microarrays to study alternative splicing events in hypoxia-associated genes in a set of ten Head and Neck Squamous Cell Carcinomas (HNSCC). These samples are a subset of 59 HNSCC collected and analysed previously [19]. The 10 samples comprised the 5 most and 5 least hypoxic samples, determined by their Hypoxia Score (HS), a gene signature derived metric of tumour hypoxia [19].

To identify hypoxia-associated genes, we developed a novel approach that increases the power of detection of differentially expressed genes by exploiting the fact that most transcripts are targeted by multiple, independent probesets. Using this approach, we identified 146 genes with significant hypoxia-related changes in exon expression across their loci, the set includes a higher number of known hypoxia-induced genes compared to the equivalent analysis on HG-U133A Plus2 arrays. To identify alternative splicing events we used a combination of the splicing index and a new metric, proposed here, based on the variation of reliability weighted fold changes (VFC). The weights are based on Detection Above the Background (DABG) scores [20],[21], which relate to the probability that the observed probeset signal is higher than the background noise distribution. We show here that the inclusion of probeset reliability information improves the detection of alternative splicing events when applied not only to our hypoxia data but also when applied to an independent dataset.

The proposed strategy identified *SLCO1B3* (Organic anion-transporting polypeptide 8 (*OATP8*), ENSG00000111700), *WDR66* (WD repeat-containing protein 66, ENSG00000158023), *COL4A6* (Collagen 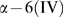 chain precursor, ENSG00000197565) and *LAMA3* (Laminin subunit 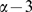 precursor, ENSG0000053747) as potentially involved in alternative splicing events related to hypoxia. The strongest evidence was for *LAMA3* which was successfully validated by RT-PCR. We also found that expression of the LAMA3-A splice variant in head and neck cancers was strongly associated with poorer survival following primary surgical treatment, showing that our methodology can be used to identify novel splicing events with prognostic significance.

## Results

### Alternative splicing in the colon cancer dataset

In order to ensure the validity of our approach, it was applied to the colon cancer sample dataset (10 paired normal-cancer samples) from Affymetrix (http://www.affymetrix.com). In [2] the dataset was analysed to identify alternative splicing events and RT-PCR validation of 49 genes (chosen based on splicing index p-values of filtered genes/probesets, manual inspection and literature information) was performed. Out of these, differential AS events in colon cancer relative to normal colon tissue were confirmed as either present or absent in 27 genes: eleven genes showed clear differential AS and 16 showed no evidence of AS. Of the remaining 22 genes, 5 showed positive results but with some ambiguity and 17 exhibited AS but were not distinctive between normal and cancerous tissues.

**Figure 1:**
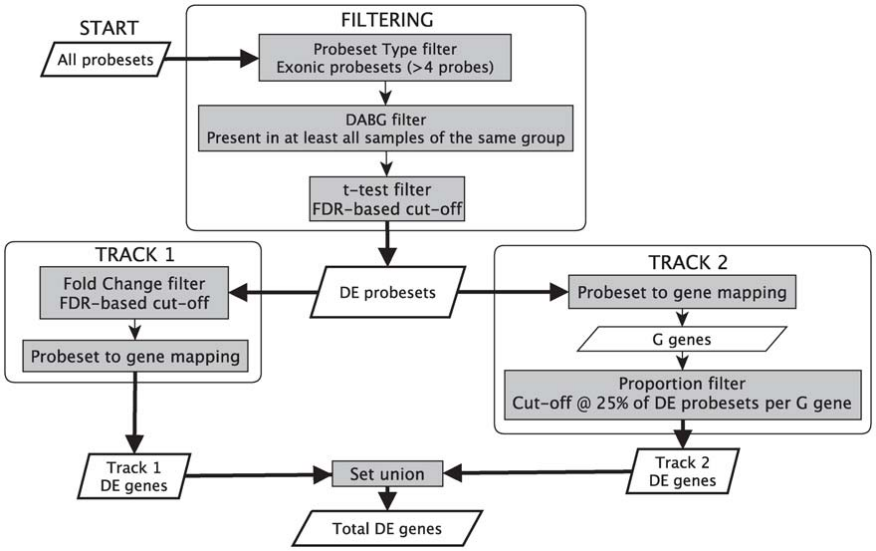
Analysis pipeline for the detection of genes with changes in expression across their loci (here referred to as differentially expressed (DE) genes) in exon array data. All array probesets go through a 3 stage filtering procedure in order to detect DE probesets. First the “Probeset filter” selects non-multiply targeting exonic probesets with 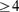 probes, then the “DABG filter” identifies the probesets present in at least all samples of the same group, finally the “t-test filter” detects probesets with a statistically-significant difference between the two groups at a 5% FDR. The resulting DE probesets are driven through two parallel tracks. Track 1: a further filter for fold change at a 5% FDR is used. This yields DE probesets with large fold changes. The probesets are mapped to genes, and these are labelled as DE genes from Track 1. Track 2: DE probesets are mapped to genes. The genes are mapped back to probesets and genes with 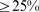 of DE probesets relative to the total non-multiply targeting exonic gene's probesets are selected and labelled as DE genes from Track 2. Total DE genes are the combined DE genes from the two tracks.

The pipeline described in Materials and Methods (Figure 1) was used to identify genes for which there were significant changes in expression in one or more exons across their length. We refer to these as differentially expressed (DE) genes, and do not at this stage consider whether expression changes are uniform across their length. We identified 1091 DE genes, 892 up-regulated in colon cancer relative to normal colon tissue and 198 down-regulated. We set the FDR cut-off of the paired t-test to 10% to include as many genes validated by RT-PCR as possible. Fifteen of the 27 genes for which AS events were confirmed by PT-PCR as either present or absent can be found in the set of DE genes obtained.

**Figure 2:**
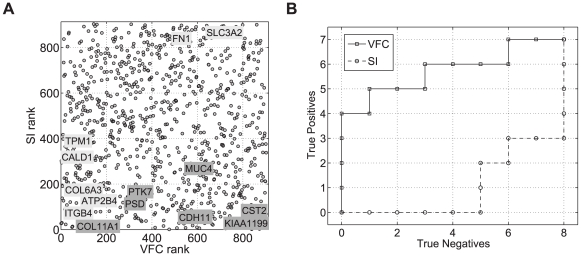
Alternative splicing in the Affymetrix colon cancer sample dataset. (A) Ranked splicing indices (SI) versus ranked VFC values. Light background: true positives, dark background: false positives. (B) ROC curve.

A plot of the ranked VFC values versus the ranked splicing indices (metrics described in Materials and Methods) of the DE genes shows that most of the genes successfully confirmed by RT-PCR are placed in the top ranks by VFC apart from *FN1* and *SLC3A2* which are in the bottom 50% (Figure 2A). Interestingly, *SLC3A2* had also a low rank in the analysis performed in [15]. Most of the genes that did not show alternative splicing events are given lower ranks by the VFC but not by the splicing index. *COL11A1* was surprisingly high in both ranks even thought it was found to have no alternative splicing event by RT-PCR validation. The inclusion of probeset reliability information in the VFC enables a better differentiation between the true and false positives (Figure 2B).

### Genes with hypoxia-induced changes in expression across their loci in the head and neck dataset

While Hypoxia causes a general down- rather than up-regulation in gene expression [22], most up-regulated genes are 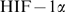 dependent [22]. This study focused on up-regulated changes, as they represent a more specific target group. The pipeline described in Materials and Methods was used to identify genes with hypoxia-induced changes in expression across their loci (i.e. hypoxia-associated genes) in the Head and Neck dataset. Essentially, the pipeline aims to find genes for which at least one exon shows a big change or for which many exons show a smaller but consistent change. The filtering stage identifies exon targeting probesets predicted to hybridize to a single locus within the genome, which are significantly differentially expressed between high and low HS samples (DE probesets). The analysis then takes place in two parallel tracks, one that identifies genes targeted by at least one DE probeset with a significantly large change (Track 1) and the other that seeks genes targeted by a high proportion of DE probesets (Track 2).

#### Filtering procedure

There are 1,411,189 probesets on the array that target 33,736 genes based on ENSEMBL annotations (Version 50). Filtering out probesets with 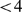 probes left 667,704 probesets targeting 28,825 genes, of which 292,040 are defined as exonic probesets. After filtering to retain probesets flagged as present (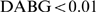) in at least 5 samples of the same group, 149,963 remained. A t-test between groups with a cut-off at an FDR of 5% yielded 3,749 DE probesets.

#### Dual track workflow

In Track 1 of the pipeline 422 DE probesets, targeting 82 genes, had a positive FC value at a 5% FDR. In Track 2 of the pipeline, up-regulated DE probesets (1,4212 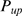 probesets) mapped to 469 genes. These genes were mapped to probesets and the proportion of 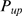 in each gene calculated. This yielded 123 genes with a high proportion (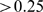) of 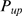. A total of 146 genes (Table S1) potentially induced under hypoxia resulted from the union of the two tracks of the procedure.

#### DE genes

The final set of DE genes was mapped on to the Gene Ontology using DAVID [23],[24]. Around one quarter of these genes are involved in processes known to be activated in tumours under hypoxia conditions [10] (e.g. cell proliferation, glycolysis, angiogenesis, cell motility and cell migration), see Table 1. This further validates the hypoxia score derived in [19] as a method of stratifying hypoxia in head and neck cancers.

**Table 1:** Hypoxia associated genes.

A	B	C	D	E	F	G	H
*SLC2A1*	*SLC3A2*	*IL1A*	*HTR7*	*YKT6*	*CDH13*	*ALDOA*	*CDH13*
*SLCO1B3*	*SLC16A1*	*TGFA*	*NRG1*	*TUBB6*	*S100A2*	*LDHA*	*ACTN1*
	*SLC7A8*	*CAV2*	*CAV1*	*CAP2*		*HK2*	*LAMA3*
	*SLC7A5*	*KCTD11*	*CAV2*	*CAV2*			
		*FOSL1*		*ACTN1*			
		*BNC1*		*TUBA1C*			
		*CDH13*		*PFN2*			
		*TCFL5*		*PLS3*			
		*CAV1*					
		*IGFBP6*					
		*PTHLH*					
		*IMPDH1*					
		*MET*					
		*NUMB*					

Genes identified as potentially induced under hypoxia that belong to processes known to be activated in tumours under hypoxia conditions. A = Anion transport, B = Carboxylic acid transport, C = Cell proliferation, D = Circulatory system process, E = Cytoskeleton organization and biogenesis, F = Endothelial cell migration, G = Glycolysis, H = Regulation of cell motility.

The list of DE genes includes 24 genes of the 99 HS genes (see Head and Neck Cancer dataset in Materials and Methods), 1 exclusively from Track 1, 14 from both tracks and 9 from Track 2 of the pipeline, highlighting the importance of Track 2. Table 2 shows the intersection between both tracks with the HS genes, and with genes previously reported in the literature to be hypoxia induced [19]. We followed an equivalent procedure to identify DE genes in the same samples arrayed in the HG-U133A Plus2 chips. We used a t-test at 5% FDR on non-multiply targeting probesets that passed the mismatch score filtering (equivalent to DABG score filtering). We obtained a total of 64 DE genes which include 12 of the 99 HS genes (Table 2). It is important to note that none of the HS/Lit genes identified by the second track are found using the HG-U133A Plus2 arrays.

**Table 2:** Hypoxia associated genes identified by exon and 3′IVT arrays.

	Exon data *T1*	Exon data *T1* & *T2*	Exon data *T2*	3′IVT data
HS genes	*IGF2BP2*	*NDUFA4L2*	*AC133461.4*	*NDUFA4L2*
		*PYGL*	*CORO1C*	*PYGL*
		*SLCO1B3*	*CNIH4*	*SLCO1B3*
		*HOMER1*	*KCTD11*	*AK3L2*
		*KRT17*	*TPD52L2*	*S100A3*
		*TANC2*		*ALC6A10P*
HS and Lit genes		*CA9*	*ALDOA*	*CA9*
		*CA12*	*LDHA*	*CA12*
		*SLC2A1*	*NDRG1*	*SLC2A1*
		*SLC16A1*		*SLC16A1*
				*SLC6A8*
				*BNIP3*
Lit genes		*F3*	*SLC3A2*	*F3*
		*TGFA*		*TGFA*
		*HK2*		
		*MET*		

Genes identified as potentially induced under hypoxia, using exon and 3′IVT arrays, which are members of the HS genes and/or genes known to be hypoxia induced in the literature (Lit genes). The genes are sort by the pipeline track (Track 1 (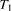) and Track 2 (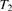)) and array platform which identified them and their membership to the set of HS genes and the set of Lit genes.

Probesets containing a single “outlier sample” tend to be rejected as DE more often in the exon arrays than in the HG-U133A Plus2 arrays because the mean difference between the high and low HS samples tends to be smaller on exon arrays (see, for example, *SLC38A5* and *HSD17B1* in Figure S1). This shift of low expression signals from U133 Plus2 to a relatively higher expression level in the exon array and the shift of high expression signal from U133 Plus2 to a relatively lower expression level in the exon array, has been identified before [2]. On the other hand, many probesets are lost to cross-hybridization in the HG-U133A Plus2 arrays, and there are not enough probesets per gene to consider the proportion of low but significant fold changes as a means to identify DE genes. Figure S2 shows the exon array data for *ALDOA*, an example of a known hypoxia-induced gene detected only by making use of the probeset multiplicity (Track 2) available in exon arrays.

### Alternative splicing in the HNSCC dataset

**Figure 3:**
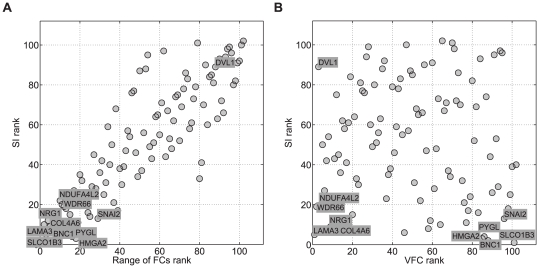
Alternative splicing in the HNSCC dataset. (A) Ranked splicing indices (SI) versus ranked range of fold change values. (B) Ranked SIs versus ranked VFC values. When the information of the DABGs is incorporated into the range of FCs, through the VFC metric, several genes high-ranked in (A) fall down the ranking in (B). Genes with bold background were further inspected manually.

**Figure 4:**
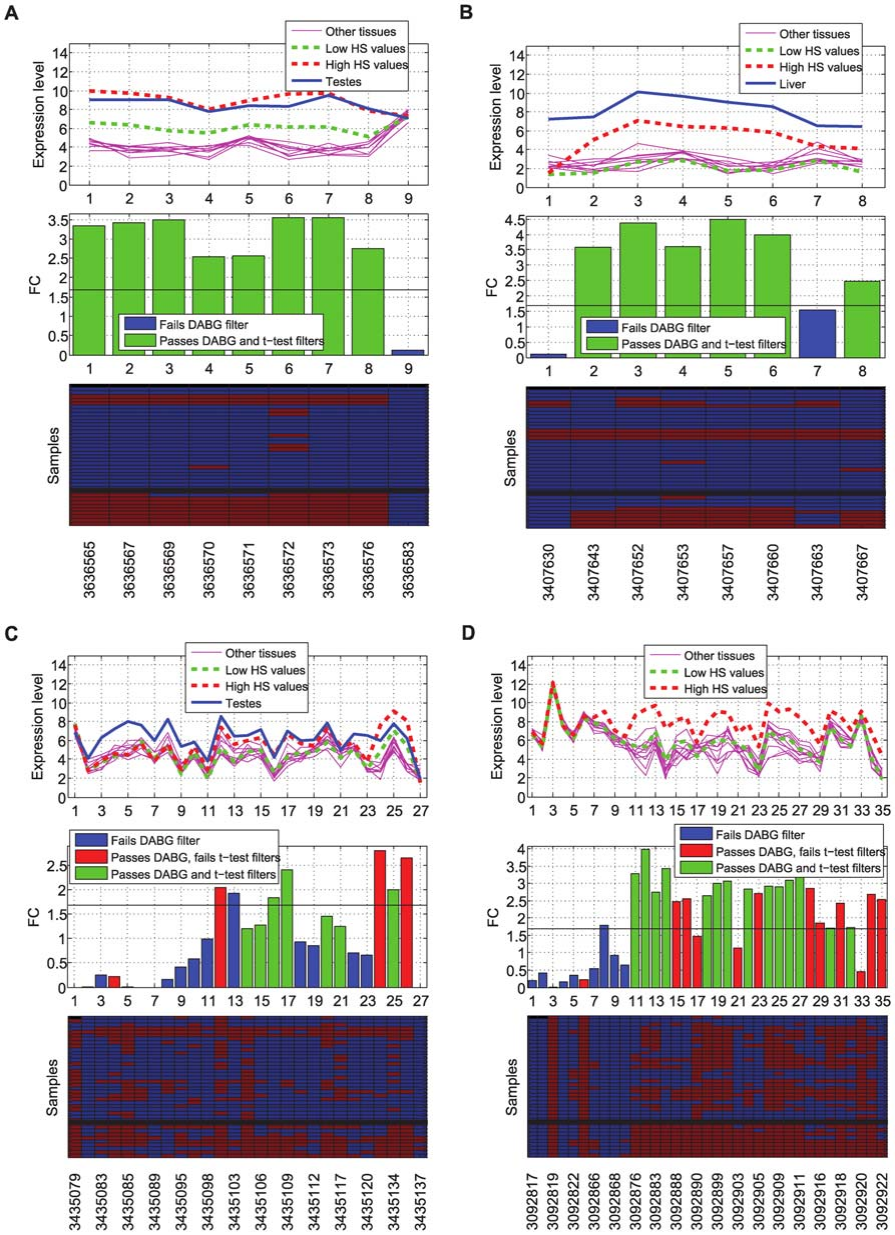
Expression of selected genes in 40 exon arrays. (A) *BNC1*, (B) *SLCO1B3*, (C) *WDR66* and (D) *NRG1* in 40 exon arrays: 10 HNSCC (5 low HS and 5 high HS) and 10 tissue types in triplicate from Affymetrix. In each figure, the top panel plots the mean expression level for each sample group (10 tissue types, low HS HNSCC and high HS HNSCC). The central panel plots the fold change value per probeset in the HNSCC dataset. The horizontal line indicates the FC threshold cut-off at 5% FDR. The bottom panel displays the DABG p-values per sample per probeset: present (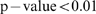) red and absent (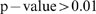) blue. The top 30 rows correspond to the 10 tissue types in triplicate, and the bottom 10 to the HNSCC samples ordered by HS score (low to high - top to bottom).

A combination of the splicing index and the VFC identified alternative splicing events in hypoxia-associated genes. Figure 3 plots the ranked splicing indices versus the ranked VFC values, before and after the inclusion of the DABG information. Six genes (*PYGL*, *BNC1*, *HMGA2*, *SLCO1B3*, *SNAI2* and *CA12*) show a high splicing index rank but low VFC rank. On further inspection, all of these have one or two probesets found to be absent in all samples, with low fold change (e.g. Figure 4A). This could indicate an alternative splicing event that is not correlated with hypoxia. We found that in five of the six genes these absent probesets were consistently absent in all replicates of the tissue panel sample dataset from Affymetrix (available at http://www.affymetrix.com/), suggesting that the absent probesets are very likely to be poorly performing probesets. An alternative splicing event was therefore considered unlikely, and these genes to be false positives. In the case of *SLCO1B3*, the three replicates of liver are present and highly expressed across all probesets, showing that the probeset absent in HNSCC is not faulty (Figure 4B). *SLCO1B3* has two known transcripts (Figure S3) and the information provided by the arrays indicates that samples with high HS express only the shorter transcript of *SLCO1B3*, while in liver the longer transcript is expressed. However, nothing can be inferred from the low HS samples because most probesets are absent in these samples. The gene in the left-top of Figure 3B (*DVL1*) is ranked high by VFC and low by splicing index. Manual comparison to the Affymetrix sample dataset, revealed that most of the FC variation is due to probesets with a low range of response.

**Figure 5:**
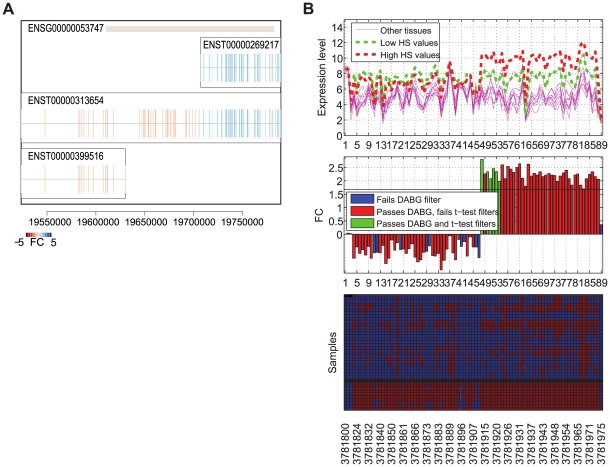
*LAMA3* transcripts, exon distribution and expression. (A) *LAMA3* transcripts and exon distribution. Fold changes per exon are indicated by the colormap. (B) Expression of *LAMA3* in 40 exon arrays: 10 HNSCC (5 low HS and 5 high HS) and 10 tissue types in triplicate from Affymetrix. The top figure plots the mean expression level for each sample group (10 tissue types, low HS HNSCC and high HS HNSCC). The middle figure plots the fold change value per probeset in the HNSCC dataset. The horizontal line indicates the FC threshold cut-off at 5% FDR. The bottom figure displays the DABG p-values per sample per probeset: present (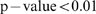) red and absent (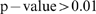) blue. The top 30 rows correspond to the 10 tissue types in triplicate, and the bottom 10 to the HNSCC samples ordered by HS score (low to high - top to bottom).

Genes with high ranks on both metrics (SI and VFC) are likely to be alternative spliced. Five genes remained at the top of the ranking of both metrics (top 0.5 quantile of the combined ranking (SI+ VFC)) after inclusion of the DABG information (*LAMA3*, *WDR66*, *NRG1*, *NDUFA4L2* and *COL4A6*). *LAMA3* is targeted by a large number of probesets, generally present across samples. Detailed examination shows that one of its three known transcripts (ENST00000269217 LAMA3-A) is differentially expressed in response to hypoxia, while the other two transcripts (ENST00000313654 LAMA3-B and ENST00000399516 LAMA3-C) show no difference (Figure 5). Expression levels of *LAMA3* in the Affymetrix sample dataset show that the low FCs in probesets targeting LAMA3-B and LAMA3-C are not due to faulty probesets. Thus, there is strong evidence that *LAMA3* is a good candidate for further validation of alternative splicing. Similar analysis provided additional support for WDR66 (Figure 4C). *NRG1* featured 3 probesets, with high absolute expression and low fold-change, that were subsequently found to show similar patterns across all samples in the Affymetrix sample dataset; suggesting that these probesets may be saturating and that *NRG1* may be a false positive (Figure 4D). *NDUFA4L2* presents a similar case to *NRG1*. *COL4A6* is targeted by a large number of probesets. These show high variation in FC with regions of similar FC values. Most probesets are absent in the five low HS samples, making it unfeasible to infer alternative splicing with respect to HS values, based on the information available. However, this information suggests that the possibility of a shorter isoform of *COL4A6* being expressed in high HS values should not be discarded.

Using our pipeline we identified *SLCO1B3*, *WDR66*, *COL4A6* and *LAMA3* as potentially involved in alternative splicing events associated with hypoxia in HNSCC.

### Validation of LAMA3

Laminin 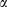 3 (*LAMA3*) forms the 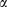 subunit of laminin-332, an extracellular glycoprotein, known to be important in cell migration and tumour invasion [25]. Laminin-332, detected by immunohistochemistry to the gamma 2 subunit, has been found at the invasive edge of squamous cell carcinomas and has been associated with a poor prognosis in a wide range of epithelial carcinomas including oral, cervical and oesophageal cancers [26]–[28]. *LAMA3* is known to be alternatively spliced [29] with the shorter transcript, Laminin 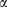 3A, encoding the protein subunit for the well characterised laminin-332.

#### Clustering and Principal Component Analysis (PCA)

We calculated the Enhanced correlation coefficient [30] of the set of probesets targeting *LAMA3* to all the probesets targeting (up- and down-regulated) DE genes. Unsupervised hierarchical clustering of the resulting correlation matrix separates *LAMA3* probesets into LAMA3-A and LAMA3-B transcripts (Figure S4). Functional annotation analysis using DAVID [23],[24] shows that the probesets with the largest contribution to the clustering of *LAMA3* probesets into the two distinctive transcripts (based on the first and second components of the PCA [31] of the correlation matrix, Figure S5 and Table S2), are significantly enriched (adjusted p-value for multiple testing 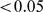) in genes involved in biological adhesion, immune system process and cell motility (Table S3).

#### qRT-PCR

qRT-PCR was carried out on RNA from the 10 original HNSCC tumours. Further RT-PCR experiments were carried out on cell lines to investigate whether expression of LAMA3-A could be induced by hypoxia in HNSCC tumour cells.

**Figure 6:**
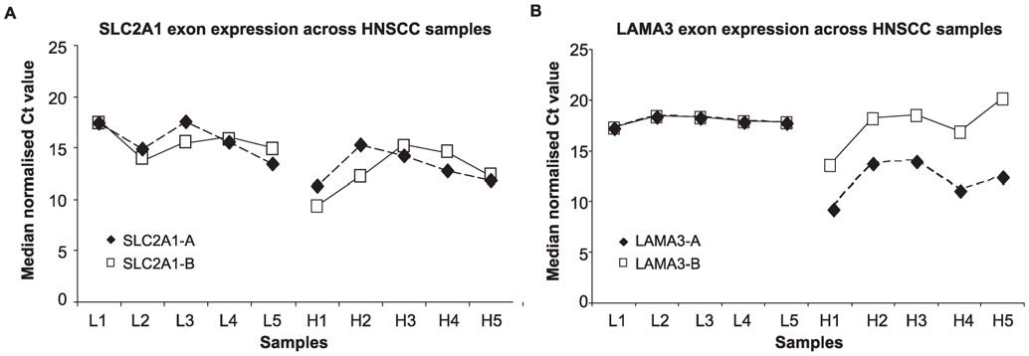
*LAMA3* in the 10 clinical HNSCC samples. The distribution of gene expression values derived from normalised quantitative RT-PCR Ct readings. A lower Ct reading indicates higher gene expression. (A) Gene expression values for primers specific to the two transcripts of *LAMA3* across 10 clinical HNSCC samples comprising 5 with low HS values (L1 to L5) and 5 with high HS values (H1 to H5). Gene expression values for LAMA3-A are significantly higher in the high HS samples compared to the low HS samples; p = 0.008 (Mann-Whitney U test). There was no significant difference in expression in LAMA3-B values between high and low HS groups. (B) Gene expression values for primers specific to two distinct regions of *SLC2A1*. Both primers show higher expression in high HS samples compared to low HS and there is no significant difference in pattern of expression of primers within samples.

There was a significant increase in expression of LAMA3-A between the 5 low and 5 high HS samples (p = 0.016), but no difference in expression of LAMA3-B between the two groups in the 10 HNSCC clinical samples (Figure 6A). *SLC2A1* (Glucose transporter type 1 (*GLUT1*), ENSG00000174640) is a 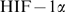 regulated, hypoxia inducible gene known to be expressed in HNSCC. As expected, the exon array data identified *SLC2A1* as being differentially expressed between the high and low HS groups but without evidence of alternative splicing. Two distinct sets of primers to *SLC2A1* which targeted different transcripts and different exons were designed to act as a positive control i.e. to show increased expression under hypoxia but without differential expression (Figure 6B). Expression of SLC2A1-A and SLC2A1-B were both significantly higher in the 5 high HS samples compared to the 5 low HS samples.

**Figure 7:**
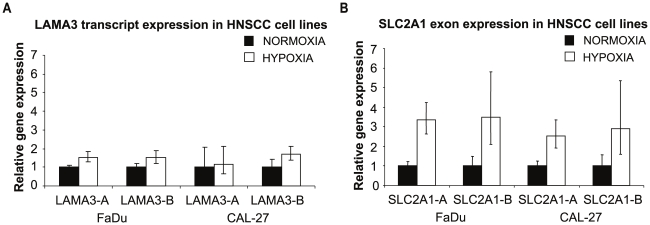
*LAMA3* in HNSCC cell lines. Relative gene expression of (A) *LAMA3* and (B) *SLC2A1* primers showing changes in expression in hypoxia relative to normoxia in CAL-27 and FaDu cell lines. At least 3 data points were collected for each of 3 independent biological repeat experiments.

In cell lines, both LAMA3-B and LAMA3-A showed no statistically significant increases in expression in response to 1% oxygen compared to atmospheric oxygen (Figure 7A). *SLC2A1* expression was consistently increased in the hypoxic samples for both assays signifying an appropriate hypoxic response in the cells (Figure 7B).

#### Outcome analysis

**Figure 8:**
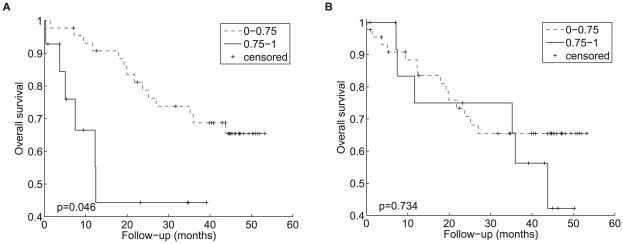
Kaplan-Meier plots of *LAMA3* expression. Kaplan-Meier plots showing overall survival of 59 HNSCC patients treated with surgery and appropriate adjuvant treatment. Patients were stratified by RNA expression (highest quartile vs. remaining three quartiles) of (A) LAMA3-A (probeset 203726_s_at) or (B) LAMA3-B (probeset 1563772_a_at).

Since HG-U133A Plus2 arrays feature four probesets to *LAMA3* (one to transcript LAMA3-A, two to LAMA3-B and one to LAMA3-B and LAMA3-C), the original microarray data from the 59 HNSCC patient series were reanalysed. Outcome data were available for this series of patients and the LAMA3-A probeset showed expression to be significantly correlated with overall survival in univariate analysis (Figure 8A), while LAMA3-B (all probesets) failed to show this correlation, see Figure 8B. Probesets targeting genes known to be hypoxia induced, such as *CA9*, *SLCO1B3* and *SLC2A1*, also failed to show a correlation with survival.

## Materials and Methods

Exon array analysis was performed using the Bioconductor package Exonmap [7],[8], which includes a variety of routines for translating between probesets, exons, genes and transcripts, defined by the annotation database X:Map [9].

### Head and neck cancer dataset

In [19] 59 Head and Neck Squamous Cell Carcinoma (HNSCCs) samples, obtained prior to any treatment at the time of primary surgery, were processed onto Affymetrix HG-U133 Plus2 arrays and a set of 99 genes up-regulated in hypoxia was obtained by analysis of genes whose *in vivo* expression clustered with the expression of 10 well-known hypoxia-regulated genes (e.g. CA9, GLUT1, and VEGF). A Hypoxia Score (HS) was defined as the median value of expression for these 99 genes (‘HS genes’). High HS values indicated higher hypoxia relative to lower values and were an adverse prognostic factor in an independent microarray dataset. HS was a continuous variable well spread across the samples (Figure S6).

Here, we first eliminated samples from the study with a high percentage of absent calls [32] by removing the top 10-th quantile of the samples ordered by number of absent calls, and then selected the 5 least and 5 most hypoxic samples as defined by the HS values. Confirmation of hypoxia status was carried out by investigating CAIX protein expression [10] in histological sections. There was a statistically significant increased CAIX expression in the samples with high HS values (p = 0.024, Figure S7). These 10 samples were then processed onto Affymetrix Human Exon 1.0ST arrays using manufacturers' standard protocols, as described in [7]. Following hybridization, we investigated the similarity in expression profiles among the 10 exon arrays. Multidimensional scaling and hierarchical clustering of the samples based on a reduced set of probesets (exonic probesets flagged present; DABG 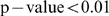 in at least half of all the samples; N = 172,204) confirmed that the samples are partitioned by high and low HS values, as expected (Figure S8). Exon array data have been deposited in NCBI's Gene Expression Omnibus [33] and are accessible through GEO Series accession number GSE18300.

### Genes with changes in expression across their loci

Figure 1 shows the analysis pipeline used to identify DE genes in exon array data. Data were first summarised using RMA [34] (there is no significant difference between RMA and PLIER in terms of alternative splicing identification [35]) and then filtered to include only exon targeting probesets, predicted to hybridize to a single locus within the genome. A DABG score filtering (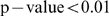 in all samples of at least one replicate set, see Text S1) and a t-test are then applied to each probeset. Here, we use the t-statistic for simplicity of implementation; however, any other suitable test could be used. An FDR[36] of 5% was used as a cut-off for statistical significance. The starting point for further analysis is then the set of differentially expressed exonic, non-multiply targeting probesets that passed the DABG score filtering. As such, it is similar to the set of DE probesets that would emerge from a standard analysis of 3′IVT arrays.

#### Two parallel tracks to identify differentially expressed genes

The availability of multiple probesets per gene can be use to increase the power of detection of DE genes beyond the sensitivity of 3′IVT arrays, by seeking a series of small but significant changes in several probesets along the gene. The alternative scenario in which a single probeset shows a larger significant change is also of interest (particularly in the context of alternative splicing).

We seek both types of events thought two parallel procedures (Figure 1). In Track 1, DE probesets with a FC cut-off defined at the 5% FDR level are selected and mapped to genes. In Track 2 of the pipeline, DE probesets (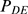) are mapped to genes. These genes are mapped back to all (4+ probe) exonic probesets (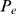) and the proportion of 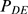 in 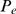 per gene is calculated. Genes with a proportion higher than 0.25 (cut-off defined using Bootstrapping; see Text S2) are selected. The total set of DE genes in the experiment results from the union of these two tracks of the procedure.

### Alternative splicing

Alternative splicing occurs as a result of the differential inclusion or exclusion of one or more exons from a gene, and can also involve the retention of intron sequence or the use of alternative 5′ and 3′ splice sites [37]. In this work we concentrated on events related to differential exon usage, therefore, introns and intergenic regions were not considered. We used the combination of two alternative splicing metrics to identify genes alternatively spliced with respect to high and low HS values: the splicing index and the VFC (Variation of reliability weighted Fold Changes). These are described in detail below.

The splicing index (SI) of probeset 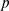 relative to gene 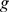 is defined as
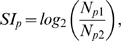
(1)where 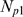 and 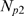 are the means of the inclusion rates of probeset 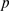 in gene 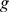 across all replicates for sample groups 1 and 2, respectively. The inclusion rate of probeset 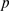, in gene 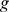, in group 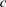, in replicate 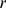 is given by
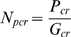
(2)where 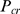 is the expression level of probeset 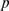 and 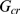 is the gene level of gene 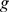. The gene level can be calculated by taking the mean or median across all exonic probesets. Overall, the splicing index is highly dependent on the gene level calculation, and is reported to work best when the gene has a large number of constitutive exons and a small number of alternative exons [38].

To calculate the VFC, the range of FCs for all exonic probesets across a gene is calculated. The range is used because it is sensitive to extremes. Alternatives, such as the coefficient of variation or the standard deviation minimise the effects of these outliers, reducing the algorithm's ability to identify single probeset changes. An obvious problem when using FCs is that each FC has different degrees of reliability specified by the DABG p-values. To incorporate this information, we centre the FC values around the median FC and weight them by the number of samples flagged present, mapped through a sigmoid transformation. We define the weight of probeset 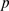 as:
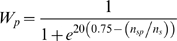
(3)where 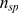 is the number of present samples of probeset 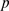 and 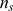 is the total number of samples. Finally, we normalise the absolute range of weighted FCs by the weighted-mean of FCs across all probesets. This normalisation is necessary to eliminate the bias resulting from the relationship between mean and range of FCs per gene. A positive correlation is observed because many genes have at least one probeset whose value is not detectable above background (high DABG p-value) in most of the samples and has a reduced difference of the mean value between the two sample groups (producing low FCs) inducing a large range of FCs across the gene by lowering the minimum FC value. The VFC of gene 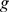 is thus:
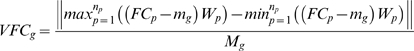
(4)where 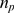 is the number of non-multiply targeting exonic probesets targeting gene 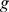, 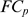 is the FC of probeset 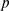, and 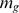 and 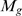 are the median and weighted-mean of FCs in gene 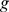.

### RT-PCR validation

#### Cell culture

Two head and neck squamous carcinoma cell lines, FaDu and CAL-27, were obtained from the American Type Culture Collection (Manassas, VA). Cells were cultured as recommended by the supplier, in Dulbecco's modified eagles media supplemented with L-glutamine (1.5 mM) and fetal bovine serum (10%). Cells were cultured at 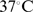 in an atmosphere of 5% 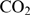 in air.

#### Hypoxia induction

Cells were plated onto glass plates and allowed to adhere for 24 hours under normoxic conditions prior to transferring to a hypoxia cabinet (Fred-Biotrace workstation) and a media change under hypoxia to hypoxia-equilibrated media. Hypoxia in this setting refers to 1% oxygen with the remainder made up of 5% 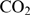 in nitrogen. Cell were trypsinised and harvested after 24 hours of exposure using hypoxia-equilibrated reagents. Cells were also cultured and harvested under normoxic conditions at the same time points. A minimum of three biological replicate experiments for each cell line was carried out.

#### RNA extraction

Total RNA was isolated using RNeasy mini kit from Qiagen, with an on-column DNase treatment (Qiagen) according to manufacturer's instructions. Concentration and purity of RNA was determined using the NanoDrop spectrophotometer (NanoDrop Technologies, Wilmington, DE). Reverse transcription was carried out using the Taqman reverse transcription reagent kit (Applied Biosystems, Cheshire, UK). One 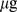 of RNA was incubated with 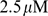 random hexamers, multiscribe enzyme and other reagents as specified by Applied Biosystems (part no. 402876, 2002). Reactions were then placed into a thermocycler (GeneAmp, PCR system 9700) and incubated at 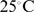 for 10 min, 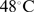 for 30 min and 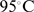 for 5 min. RNA was extracted from HNSCC samples as described previously [19]. RNA from clinical samples was reverse transcribed using the same reagents as for cell lines with the exception of using oligo dT primers instead of random hexamers.

#### Selection of validation candidate genes

Preference was given to genes in which exon expression patterns matched that of known splice variants combined with good probeset reliability. *LAMA3* provided the most consistent evidence of hypoxia induced alternative splicing and was chosen for validation. Because the two transcripts of *LAMA3* varied only at their 5′ end it was not possible to distinguish them using RT-PCR based methods so quantitative real time PCR (qRT-PCR) was used. *SLC2A1* is a well known hypoxia induced gene which did not appear to be alternatively spliced in the exon array data or using RT-PCR analysis (Figure S9). Primers to the 5′ and 3′ ends of this gene were designed to act as control assays.

#### Primer design

qRT-PCR primers were designed using the Exiqon universal ProbeLibary systems available at www.roche-applied-science.com (Roche, Basal, Switzerland). Primer oligonucleotides were obtained from MWG (Ebersberg, Germany). Primer sequences are shown in Table 3. Primers to endogenous reference genes were designed using the same system (Figure S10). Primers were screened for potential single nucleotide polymorphisms or pseudogene binding. Satisfactory primer efficiencies were determined by standard curves of known relative concentrations of cell line cDNA.

**Table 3:** Primer sequences for *LAMA3* and *SLC2A1*.

	Forward	Reverse	Transcript	Exon	UP	AL
LAMA3-A	TGCAAGCGAG	CAAGCCTTTAT	ENST00000269217	1–2	#20	74 nt
	TTATGTGGAG	GATCCCGATA				
LAMA3-B	CCAGGAATAT	GGGAGCAGCA	ENST00000313654	20–21	#29	65 nt
	GGGTTGCTTG	CCAGGTAAT				
SLC2A1-A	GAGCCCAGCA	TGATGACTCCA	ENST00000372500	1–2	#52	97 nt
	GCAAGAAG	GTGTTGTAGCC	ENST00000372501			
SLC2A1-B	GCCAGCCAAA	GACTCACACTT	ENST00000372501	10	#17	69 nt
	GTGACAAGAC	GGGAATCAGC	ENST00000397019			

Primer sequences for LAMA3-A (transcript ENST00000269217), LAMA3-B (transcript ENST00000313654), SLC2A1-A (transcripts ENST00000372500 and ENST00000372501) and SLC2A1-B (transcripts ENST00000372501 and ENST00000397019). UP = UPL probe and AL = amplicon length.

#### Real-Time PCR

Expression analysis by qRT-PCR was carried out using an ABI 7900 sequence detector (ABI Biosciences, Warrington, UK). qRT-PCR assays were carried out in 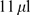 reaction volumes in 384 well plates containing 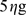 cDNA in 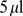, 5 pmol primer oligonucleotide, 5 pmol of ProbeLibrary probe, and 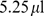 Taqman mastermix (ABI). Pipetting of 384 well plates was performed using a 5070 epMotion pipetting robot (Eppendorf, Hamburg, Germany). PCR conditions were standardized with temperatures of 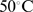 for 2 min, 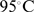 for 10 min, followed by 40 cycles of 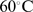 for 1 min and 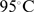 for 15 s. Threshold cycles were determined automatically using SDS2.1 software.

#### Determination of endogenous reference genes

A panel of 10 reference genes was used, comprising five commonly used reference genes and five genes identified as being consistently expressed across the 59-sample microarray dataset. Using GeNorm software [39] 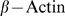 and *RPL11* were selected as housekeeper control genes for the cell lines, while *GNB2* and *RPL24* were used for the clinical samples. Reference genes were evaluated on samples from both cell lines cultured in hypoxia and normoxia. Samples were tested in triplicate and median values calculated.

#### Clinical samples

Tumour samples were collected as part of an ethically approved study from patients undergoing potentially curative resection of HNSCC in Manchester and Oxford. Sample processing and RNA extraction has been described previously [19]. Sample demographics are summarised in Table S4.

#### Statistical analysis

Clinical RT-PCR data were expressed as Ct values normalised to the expression of the validated endogenous reference genes. The difference in expression between the 5 high HS and 5 low HS samples was calculated using a Mann-Whitney test based upon the ranking of the median expression of each sample. When the number of undetermined values meant that samples were tied, ranking was done according to the number of technical replicates leading to a determined value.

Cell line RT-PCR data were analysed according to the 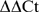 method. In short, the mean of triplicate measures of each gene target was normalized using the geomean of the two selected endogenous reference genes. Relative expression of the normalized values for hypoxic samples relative to the comparable normoxic samples were determined and the relative gene expression value determined using the 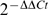 method [40].

## Discussion

There are several publications showing a good correspondence between fold change values in the Exon and the 3′IVT arrays (e.g. [2],[6],[41]). These comparisons are usually done on a reduced set of genes with overlapping probeset locations. However, these analyses have not compared the relative ability of the platforms to detect differential expression in a supervised analysis. In part this is because the main focus in exon array analysis is the study of alternative splicing. Our work highlights how the analysis of differential expression is enhanced by using the probeset multiplicity offered by exon arrays.

We took a novel approach to the handling of DABG p-values in the identification of alternative splicing events. Typically, when filtering is performed at all, probesets absent in more than a predefined number of samples are filtered out. We retain all exonic probesets per gene when calculating the alternative splicing metric, but weight their contribution by the number of present samples. This approach allows a continuous scoring of the reliability of the probesets based on the DABG p-values across the samples, avoiding an abrupt ‘in-or-out’ filtering. We also found that on a number of occasions, a single probeset was responsible for a gene being flagged as alternatively spliced, but that on further investigation, that probeset showed little change across a set of independent experiments, leading us to conclude that the findings were likely to be spurious.

We first tested our methodology on a sample dataset for which predicted alternative splicing events where explored by RT-PCR and we were able to confirm that the inclusion of probeset reliability information in the VFC metric enables a better differentiation between the true and false positives. We then used the method to analyse our Head and Neck dataset and four hypoxia-associated alternative spliced candidates were identified (*SLCO1B3*, *WDR66*, *COL4A6* and *LAMA3*). We further analysed and validated *LAMA3*, which showed the strongest evidence. The finding was successfully confirmed by RT-PCR and an informed re-analysis of the original microarray data allowed probes matched to the *LAMA3* transcripts to be identified and a hypoxia-associated, splice variant dependent prognostic relationship with outcome to be determined. Antibodies specific to the different splice variants of LAMA3 were not available, precluding analysis of the different LAMA3 transcripts at the protein level, but identification of the prognostic significance of expression of the LAMA3-A versus LAMA3-B splice variant illustrates the potential for alternatively spliced transcripts to act as biomarkers of disease. The additional information provided by splicing data has the potential to lead to improved specificity for particular tissues or conditions, over assays that do not discriminate between splice variants. This also emphasizes the importance of identifying specific splice variants when interpreting gene expression data.

Cell line experiments at 1% hypoxia failed to demonstrate convincing hypoxic induction of LAMA3-A, with only low levels of hypoxia induced expression seen, despite confirmation of a transcriptional hypoxic response through *SLC2A1* (*GLUT1*) expression. Our initial stratification of samples for exon array analysis was based upon the expression of a gene signature of hypoxia associated genes; direct measurement of hypoxia in these tumours *in vivo* was not performed. Instead, additional confirmation of hypoxia status was carried out by investigating CAIX protein expression in histological sections. CAIX expression was indeed elevated in samples with high HS values supporting the use of the hypoxia associated gene expression score as a surrogate marker for tumour hypoxia, and supporting the hypothesis that differential LAMA3-A expression is related to tumour hypoxia. It may be that greater or more prolonged hypoxia, lower pH or lower glucose levels are required for *LAMA3* induction in cell lines or that this simply represents differences between cell line experiments and the situation in tumour. *LAMA3* has independently been shown to be 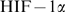 regulated in human keratinocyte wound response experiments, using Cobalt Chloride to induce 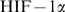 in this case instead of direct hypoxia exposure, and to have a hypoxia response element associated with the promoter for LAMA3-A [42]. This represents the likely mechanism underlying any hypoxia associated differential expression of this transcript. An earlier study however had shown decreased laminin-332 expression in human keratinocytes in response to 0.2% or 2% hypoxia exposure [43]. Laminin-332 is known to interact with several components of the extracellular matrix; particularly its interaction with Collagen VII has been shown to be vital for tumour development in skin cancers [44]. Our data would suggest that *LAMA3* induction in HNSCC tumours is influenced by hypoxia but the lack of expression seen in our HNSCC cell lines implies that expression may also be dependant upon other factors found in tissues but not in cell culture. Hypoxia is inherently associated with treatment resistance and a more aggressive tumour phenotype [10]. It is possible that LAMA3-A expression is dependent upon factors related to this relationship rather than being independently hypoxia inducible. Whilst the exact pathways involved in the expression of this transcript are unclear this study emphasizes the importance of identifying individual transcript expression in future biomarker research.

## Supporting Information

Figure S1 Probeset expression in HG-U133A Plus2 arrays and exon arrays. Genes (probesets) containing a single “outlier sample” tend to be rejected as differentially expressed more often in the exon arrays than in the HG-U133A Plus2 arrays because the mean difference tends to be smaller in exon arrays. SLC38A5 and HSD17B1 as examples of this phenomenon.
(0.30 MB EPS)

Figure S2 ALDOA expression in 10 exon arrayed HNSCC samples. The top panel shows the mean expression level for each group (Low and High HS) and “error bars” the maximum and minimum value for each group. The bottom panel plots the fold change value per probeset; the significantly differentially expressed probesets are well below the FC cut-off level (shown by the horizontal black line), i.e. have low fold changes.
(0.36 MB EPS)

Figure S3 SLCO1B3 exon array information. SLCO1B3, also known as (OATP8) organic anion-transporting polypeptide 8, has two known transcripts in the ENSEMBL database: ENST00000381545 and ENST00000261196. (A) Gene structure in terms of transcripts and exons; exons coloured by fold change. (B) Expression of SLCO1B3 in 40 exon arrays: 10 tissue types in triplicate from Affymetrix and 10 HNSCC (5 low HS and 5 high HS). In each panel, the top figure plots the mean expression level for each sample group (10 tissue types, low HS HNSCC and high HS HNSCC). The middle figure plots the fold change value per probeset in the HNSCC dataset. The bottom figure displays the DABG p-values per sample per probeset: present (DABG<0.01) red and absent (DABG>0.01) green. The top 30 rows correspond to the 10 tissue types in triplicate, the bold line separates the bottom 10 HNSCC samples ordered by HS score (high to low bottom-top).
(0.05 MB PDF)

Figure S4 Correlation matrix analysis of LAMA3 probesets. Clustering of the correlation matrix of LAMA3 probesets (transcripts LAMA3-A and LAMA3-B) versus probesets targeting (up- an down-regulated) DE genes. Heatmap of correlations ordered by hierarchical clustering in both dimensions. Horizontal axis correspond to the probesets targeting DE genes and vertical axis to the probesets targeting LAMA3 (LAMA3-A and LAMA3-B transcripts). The top cluster in the vertical axis (left) corresponds to the probesets targeting LAMA3-B only, while the bottom cluster corresponds to probesets targeting LAMA3-B and LAMA3-A.
(0.91 MB EPS)

Figure S5 PCA of the correlation matrix of LAMA3 probesets. First and second component of the PCA of the correlation matrix of LAMA3 probesets (transcripts LAMA3-A and LAMA3-B) versus probesets targeting DE genes. LAMA3 probesets separate well into the two distinct transcripts.
(0.02 MB EPS)

Figure S6 Hypoxia Score (HS) distribution. HS is a continuous variable well-spread across the samples. The bars represent the HS values for the 59 HNSCCs (order by increasing HS values).
(0.02 MB EPS)

Figure S7 Confirmation of hypoxia status in the HNSCC samples was carried out by investigating CAIX protein expression in histological sections. There was a statistically significant increased CAIX expression in the samples with high HS values (p = 0.024). Paraffin blocks were unavailable for samples L3 and H2.
(0.01 MB PDF)

Figure S8 Clustering of the 10 HNSCC exon arrays. Ten HNSCC samples, five with low (L1, L2, …) and 5 with high (H1, H2, …) HS values. (a) Multidimensional Scaling (MDS) of the samples - distances among the samples reflect similarity based on correlation, (b) Hierarchical clustering of the samples (correlation-based, complete linkage).
(0.24 MB EPS)

Figure S9 PCR products from RT-PCR of SLC2A1. Gel showing PCR products from RT-PCR of a long transcript for SLC2A1, using primers SLC2A1-A forward and SLC2A1-B reverse (predicted length 1479 bp). No smaller PCR fragments were seen indicating no alternative splicing in this transcript. An increase in intensity can be seen in the hypoxic sample showing hypoxic induction of the gene. Primers for SLC2A1-A and Beta-Actin were also run as a control.
(0.58 MB EPS)

Figure S10 Primers to endogenous reference genes. M-values obtained from use of the GeNorm applet. GeNorm assesses the pair wise distribution of endogenous reference genes, identifying the pair of genes with the most stable geometric mean (lowest M-value). (a) To determine endogenous reference genes for cell lines, cDNA was prepared from both HNSCC cell lines across hypoxic and normoxic time points using 1% hypoxia. (b) To determine reference genes for use in clinical samples, cDNA from 40 HNSCC samples were analysed using RT-PCR. All qRT-PCR assays were carried out using the same method as described in the main text and were carried out on the same 384 well PCR card. Genes giving undetermined readings were excluded from analysis.
(0.37 MB EPS)

Table S1 Identified hypoxia associated genes.
(0.27 MB EPS)

Table S2 Genes targeted by probesets with the largest contribution to the clustering of the transcripts in the PCA (Figure S5), highly correlated (top quartile) to the set of probesets targeting LAMA3-A.
(0.28 MB EPS)

Table S3 Lists the biological processes significantly enriched in the gene list shown in Table S2.
(0.25 MB EPS)

Table S4 Demographics of High HS and Low HS groups.
(0.22 MB EPS)

Text S1 Filtering by DABG scores and Bootstrapping
(0.03 MB PDF)

## References

[1] Clark F, Thanaraj TA (2002). Categorization and characterization of transcript-confirmed consitutively and alternatively spliced introns and exons from human.. Human Molecular Genetics.

[2] Gardina PJ (2006). Alternative splicing and differential gene expression in colon cancer detected by a whole genome exon array.. BMC Genomics.

[3] Johnson J, Castle J, Garrett-Engele P, Kan Z, Loerch P (2003). Genome-wide survey of human alternative pre-mRNA splicing with exon junction microarrays.. Science.

[4] Hanahan D, Weinberg R (2000). The hallmarks of cancer.. Cell.

[5] Venables-2006 (2006). Unbalanced alternative splicing and its significance in cancer.. Bioessays.

[6] Zhang X, Liu G, Lengurg ME, Spira A (2007). Comparison of smoking-induced gene expression on Affymetrix Exon and 3′-based expression arrays.. Genome Informatics.

[7] Okoniewski MJ, Yates T, Dibben S, Miller CJ (2007). An annotation infractructure for the analysis and interpretation of Affymetrix exon array data.. Genome Biology.

[8] Okoniewski MJ, Miller CJ (2008). Comprehensive analysis of Affymetrix exon arrays using BioConductor.. PLoS Computational Biology.

[9] Yates T, Okoniewski MJ, Miller CJ (2007). X:Map: annotation and visualization of genome structure for Affymetrix exon array analysis.. Nucleic Acids Research..

[10] Harris A (2002). Hypoxia - a key regulatory factor in tumour growth.. Nature Reviews Cancer.

[11] Harper SJ, Bates DO (2008). VEGF-A splicing: the key to anti-angiogenic therapeutics?. Nature Reviews Cancer.

[12] Barathova M, Takacova M, Holotnakova T, Gibadulinova A, Ohradanova A (2008). Alternative splicing variant of the hypoxia marker carbonic anhydrase IX expressed independently of hypoxia and tumour phenotype.. Br J Cancer.

[13] Srinivasan K (2005). Detection and measurement of alternative splicing using splicing-sensitive microarrays.. Methods.

[14] Shah S, Pallas JA (2009). Identifying differential exon splicing using linear models and correlation coefficients.. BMC Bioinformatics.

[15] Purdom E, Simpson KM, Robinson MD, Conboy JG, Lapuk AV (2008). FIRMA: a method for detection of alternative splicing from exon array data.. Bioinformatics.

[16] Xing Y, Stoilov R, Kapur K (2008). Mads: A new and improved method for analysis of differential alternative splicing by exon-tiling microarrays.. RNA.

[17] French PJ, Peeters J, Horsman S, Duijm E, Siccama I (2007). Identification of differentially regulated splice variants and novel exons in glial brain tumors using exon expression arrays.. Cancer Research.

[18] Schutte M, Elstrodt F, Bralten LBC, Nagel JHA, Duijm E (2008). Exon expression arrays as a tool to identify new cancer genes.. PLoS one.

[19] Winter SC, Buffa FM, Silva P, Miller C, Valentine HR (2007). Relation of a hypoxia metagene derived from head and neck cancer to prognosis of multiple cancers.. Cancer Res.

[20] Clark TA, Schweitzer AC, Chen TX, Staples MK, Lu G (2007). Discovery of tissue-specific exons using comprehensive human exon microarrays.. Genome Biology.

[21] Affymetrix. Exon array background correction,Affymetrix Whitepaper, available: http://www.affymetrix.com/support/technical/whitepapers/exon_background_correction_whitepaper.pdf

[22] Greijer AE, van der Greop P, Kemming D, Shvarts S, Semenza GL (2005). Up-regulation of gene expression by hypoxia is mediated predominantly by hypoxia-inducible factor 1 (HIF-1).. Journal of Pathology.

[23] Huang DW, Sherman BT, Lempicki RA (2009). Systematic and integrative analysis of large gene lists using DAVID Bioinformatics Resources.. Nature Protocols.

[24] Deniss GJ, Sherman BT, Hosack DA, Yang J, Gao W (2003). DAVID: Database for Annotation, Visualization, and Integrated Discovery.. Genome Biology.

[25] Marinkovich MP (2008). Laminin 332 in squamous-cell carcinoma.. Nat Rev Cancer.

[26] Ono Y, Nakanishi Y, Ino Y, Niki T, Yamada T (1999). Clinicopathologic significance of laminin-5 γ 2 chain expression in squamous cell carcinoma of the tongue.. Cancer.

[27] Skyldberg B, Salo S, Eriksson E, Aspenblad U, Moberger B (1999). Laminin-5 as a marker of invasiveness in cervical lesions.. J Natl Cancer Inst.

[28] Yamamoto H, Itoh F, Iku S, Hosokawa M, Imai K (2001). Expression of the *γ* 2 chain of laminin-5 at the invasive front is associated with recurrence and poor prognosis in human esophageal squamous cell carcinoma.. Clin Cancer Res.

[29] Ferrigno O, Virolle T, Galliano MF, Chauvin N, Ortonne JP (1997). Murine laminin α 3a and α 3b isofrom chains are generated by usage of two promoters and alternative splicing.. J of Biological Chemistry.

[30] Moller-Levet CS, West C, Miller CJ (2007). Exploiting sample variability to enhance multivariate analysis of microarray data.. Bioinformatics.

[31] Jolliffe IT (1986). Principal Component Analysis..

[32] Liu WM, Mei R, Di X, Ryder T, Hubbell E (2002). Analysis of high density expression microarrays with signed-rank call algorithms.. Bioinformatics.

[33] Edgar R, Domrachev M, Lash AE (2002). Gene Expression Omnibus: NCBI gene expression and hybridization array data repository.. Nucleic Acids Research.

[34] Irizarry RA (2003). Exploration, normalization, and summaries of high density oligonucleotide array probe level data.. Biostatistics.

[35] Beffa CD, Cordero F, Calogero RA (2008). Dissecting an alternative splicing analysis workflow for GeneChip (R) Exon 1.0 ST Affymetrix arrays.. BMC Genomics.

[36] Benjamini Y, Hochberg Y (1995). Controlling the False Discovery Rate: a practical and powerful approach to multiple testing.. J R Statis Soc.

[37] Modrek B, CL (2002). A genomic view of alternative splicing.. Nature Genetics.

[38] Affymetrix. Identifying and validating alternative splicing events,Technical Note, available: http://www.affymetrix.com/support/technical/technotes/id_altsplicingevents_technote.pdf

[39] Vandesompele J, de Preter K, Pattyn F, Poppe B, van Roy N (2002). Accurate normalization of real-time quantitative RT-PCR data by geometric averaging of multiple internal control genes.. Genome Biology.

[40] Livak KJ, DST (2001). Analysis of relative gene expression data using real-time quantitative pcr and the 2^−ΔΔ*CT*^ method.. Methods.

[41] Robinson MD, Speed TP (2007). A comparison of Affymetrix gene expression arrays.. BMC Bioinformatics.

[42] Fitsialos G, Bourget I, Augier S, Ginouves A, Rezzonico R (2008). HIF1 transcription factor regulates laminin-332 expression and keratinocyte migration.. Journal of cell science.

[43] O'Toole EA, Marinkovitch MP, Peavey CL, Ameiva M, Furthmayr H (1997). Hypoxia increases human keratinocyte motility on connective tissue.. Journal of Clinical Investigation.

[44] Ortiz-Urda S, Garcia J, Green CL, Chen L, Lin Q (2005). Type VII collagen is required for Ras-driven huma epidermal tumorigenesis.. Science.

