# Supplementary figures and images for: Exon Array Analysis of Head and Neck Cancers Identifies a Hypoxia Related Splice Variant of LAMA3 Associated with a Poor Prognosis
Source: PLoS Comput Biol. 2009 Nov 20;5(11):e1000571. doi: 10.1371/journal.pcbi.1000571 (PMC2773424; doi:10.1371/journal.pcbi.1000571)

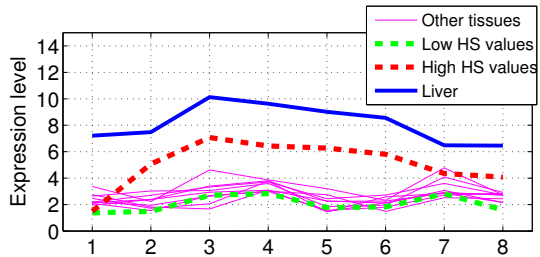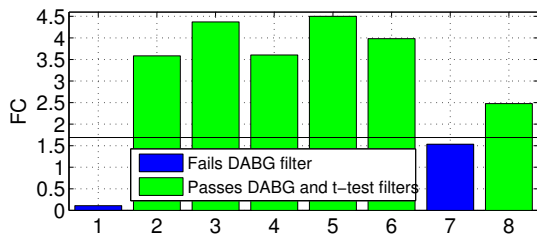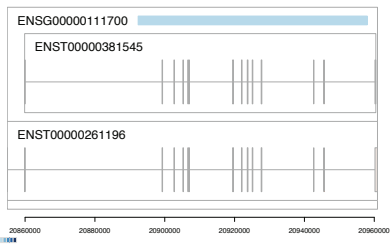

(a) Transcripts

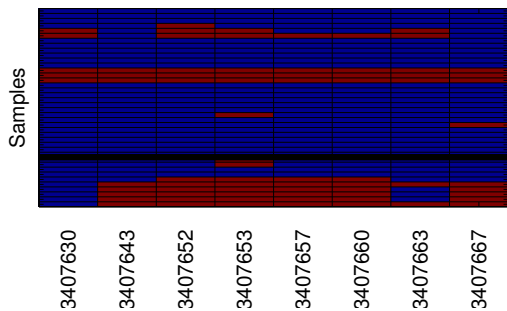

(b) Exon array information of SLCO1B3

Supplement: Figure S3 — SLCO1B3 exon array information. SLCO1B3, also known as (OATP8) organic anion-transporting polypeptide 8, has two known transcripts in the ENSEMBL database: ENST00000381545 and ENST00000261196. (A) Gene structure in terms of transcripts and exons; exons coloured by fold change. (B) Expression of SLCO1B3 in 40 exon arrays: 10 tissue types in triplicate from Affymetrix and 10 HNSCC (5 low HS and 5 high HS). In each panel, the top figure plots the mean expression level for each sample group (10 tissue types, low HS HNSCC and high HS HNSCC). The middle figure plots the fold change value per probeset in the HNSCC dataset. The bottom figure displays the DABG p-values per sample per probeset: present (DABG<0.01) red and absent (DABG>0.01) green. The top 30 rows correspond to the 10 tissue types in triplicate, the bold line separates the bottom 10 HNSCC samples ordered by HS score (high to low bottom-top). (0.05 MB PDF) [file pcbi.1000571.s003.pdf]

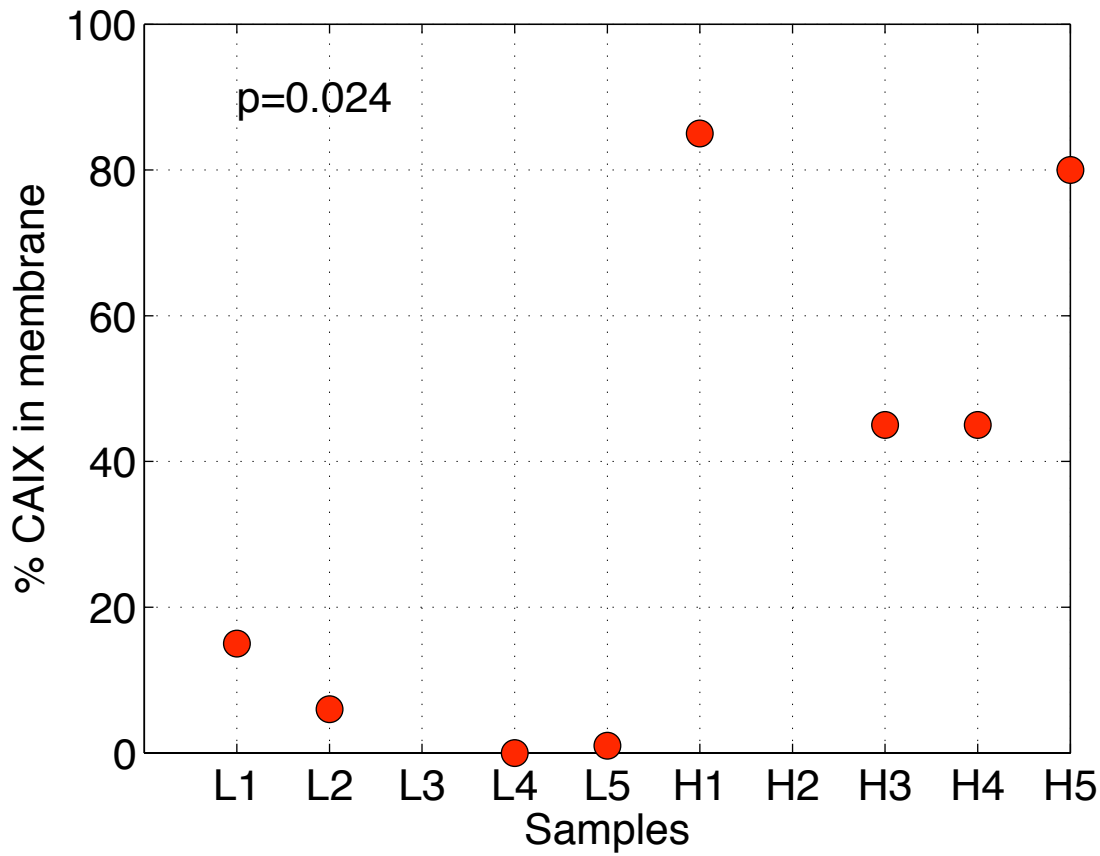

Supplement: Figure S7 — Confirmation of hypoxia status in the HNSCC samples was carried out by investigating CAIX protein expression in histological sections. There was a statistically significant increased CAIX expression in the samples with high HS values (p = 0.024). Paraffin blocks were unavailable for samples L3 and H2. (0.01 MB PDF) [file pcbi.1000571.s007.pdf]
