# Supplementary material for: Exon Array Analysis of Head and Neck Cancers Identifies a Hypoxia Related Splice Variant of LAMA3 Associated with a Poor Prognosis
Source: PLoS Comput Biol. 2009 Nov 20;5(11):e1000571. doi: 10.1371/journal.pcbi.1000571 (PMC2773424; doi:10.1371/journal.pcbi.1000571)
Supplement: Text S1 — Filtering by DABG scores and Bootstrapping (0.03 MB PDF) [file pcbi.1000571.s015.pdf]

Supporting Text

S1 - Filtering by DABG scores

Detection Above BackGround (DABG) scores compare the expression level of the probes within a probeset to a reference pool of GC-content matched background probes that are designed with sequences that are not expected to match with 10% identity to any sequence within the genome. These probes are used to measure non-specific hybridization and the DABG score to assess the signal to noise ratio for each probeset. DABG scores are reported as p-values.

There are typically two variables that influence dataset filtering using DABG p-values. These are: the minimum number of samples declared present (f_s), and the p-value threshold used to declare a sample present (f_p). DABG calls have been found to be useful in removing poorly performing probesets prior to analysis. Here, we found that the proportion of significant DE probesets (based on t-test p-values) at a given False Discovery Rate (FDR) increases as f_p decreases and f_s increases (data not shown). Thus, the more stringent the DABG filtering, the less noisy the resulting dataset. Here, we set f_s such that at least all samples must be called present in the same group, and $f_p = 0.01$.

S2 - Bootsrapping

In Track 2 of the pipeline, genes having a proportion of DE probesets (relative to the total gene's exonic probesets) higher than a cut-off value are identified as DE. The cut-off value was set by bootstrapping the same number of DE probesets (3,749) from the set of pre-filtered probesets (149,963), up-regulated probesets are selected and further labelled as P-up probesets. In 100 repetitions, the proportion of P-up probesets relative to the total gene's exonic probesets stabilized at 20% with proportion values always lower than 25%.
